# The application research of xTAG GPP multiplex PCR in the diagnosis of persistent and chronic diarrhea in children

**DOI:** 10.1186/s12887-020-02206-6

**Published:** 2020-06-26

**Authors:** Chunli Wang, Xiaoying Zhou, Mengshu Zhu, Hanjun Yin, Jiamei Tang, Yan Huang, Bixia Zheng, Yu Jin, Zhifeng Liu

**Affiliations:** 1 Nanjing Key Laboratory of Pediatrics, Children’s Hospital of Nanjing Medical University, Nanjing, 210008 China; 2 Department of Gastroenterology, Children’s Hospital of Nanjing Medical University, 72 Guangzhou Road, Nanjing, 210008 Jiangsu Province China

**Keywords:** Persistent diarrhea, Chronic diarrhea, Nucleic acid amplification techniques, Viruses, Bacteria, Parasites

## Abstract

**Background:**

Persistent and chronic diarrhea is difficult to treat, and infection is still the main cause. In this study, we investigate the application value of xTAG gastrointestinal pathogen panel (xTAG GPP) multiplex PCR in the early diagnosis of persistent and chronic diarrhea in children and to understand the epidemiology of intestinal diarrhea pathogens.

**Methods:**

One hundred ninety-nine specimens were collected from Nanjing Children’s Hospital Affiliated to Nanjing Medical University (Nanjing, China). We compared the xTAG GPP multiplex PCR assay with traditional methods (culture, rapid enzyme immunoassay chromatography, and microscopic examination) and performed a statistical analysis.

**Results:**

The positive rate of the xTAG GPP multiplex PCR assay of diarrhea specimens from 199 patients was 72.86% (145/199). The virus detection rate was 48.7%, and rotavirus A was the most common organism detected (34.67%), concentrated in winter, and was common in children. The second most common organism detected was norovirus GI/GII (20.6%). The positive rate of this bacteria was 40.2%, and *Campylobacter* (22.11%, 44/199) was most frequently detected. *C. difficile* toxins A/B and *Salmonella* was detected in 44 and 17 samples, respectively. Infections with *Shigella* occurred 4 times, and *E. coli* O157 was only detected once. Three samples were parasitic (1.51%), two samples were positive for *Entamoeba histolytica*, and one was positive for *Cryptosporidium*. Adenovirus 40/41, STEC, ETEC, *Giardia*, *Yersinia enterocolitica* and *Vibrio cholerae* were not detected. In total, 86 (43.2%) infected specimens with a single pathogen were detected. There were 59 coinfections (29.65% of the samples) of viruses and/or bacteria and/or parasites. Coinfections involved 49 double infections (24.62%), 9 triple infections (4.52%) and 1 quadruple infections (0.5%). Norovirus GI/GII was found to have the highest involvement, with 32 coinfections (16.08%).

**Conclusion:**

The xTAG GPP multiplex PCR assay is simple, sensitive, and specific and can be used as a quick way to diagnose persistent and chronic diarrhea in children.

## Background

Diarrhea continues to be a health burden worldwide, especially in children living in developing countries. It is estimated that in these regions, it is responsible for 2.5 million infant deaths annually, with a mortality rate of 4.9 per 1000 children and an annual incidence of 3 episodes per child among children under 5 years of age [1, 2]. Most of the diarrheal illnesses are acute, lasting no more than 7 days; however, approximately 3–19% of the acute episodes last more than two weeks, which is called persistent and chronic diarrhea [3]. Persistent and chronic diarrhea cases are difficult to treat and their treatment cost is higher, and a case fatality rate as high as 60% has been reported [4]. It is more important to determine the cause of chronic diarrhea by a systematic approach because it can provide the most suitable therapy and give a good prognosis. The causes of chronic diarrhea are divided into infectious and noninfectious etiologies. In developed countries, the incidence of noninfectious-based diseases (food allergies, enteropathy or inflammation) is increasing. However, in developing and industrialized countries, the most common and most important cause of persistent and chronic diarrhea is still enteric infection [5, 6]. It is crucial for timely and effective treatment of infectious diarrhea in the rapid identification of pathogens because appropriate antimicrobial therapy and/or isolation measures to prevent the spread of infectious agents to healthy people can shorten the disease and reduce some bacteria and parasite infection incidence and can help reduce invasive infections [7, 8]. The gold standard for the diagnosis of infectious chronic diarrhea pathogens is culturing pathogens, but this method takes a long time (72 h) and requires more fecal sample [9]. In recent years, the development of faster and more sensitive molecular tests that can detect various pathogenic agents of bacteria, viruses and parasites might improve the etiological diagnosis of diarrhea pathogens [9–11].

The Luminex® Corporation has developed a new qualitative bead-based multiplexed molecular diagnostic test, the xTAG gastrointestinal pathogen panel (xTAG GPP), that can be performed directly on stool samples to detect and identify 15 pathogens in a timely manner: Adenovirus 40/41, *Campylobacter*, *Clostridium difficile*, *Cryptosporidium*, *Entamoeba histolytica*, enterotoxigenic *Escherichia coli* (ETEC), *E. coli* O157, Shiga-like toxin-producing *E. coli* (STEC), *Shigella*, *Salmonella*, *Giardia*, norovirus GI/GII, rotavirus A, *Vibrio cholerae* and *Yersinia enterocolitica* [12]. The clinical manifestation of xTAG GPP was recently evaluated in many infectious gastroenteritis cases, the sensitivity and specificity is better in xTAG GPP than in traditional methods [13, 14].

Thus, the purpose of our study was to explore the distribution of enteropathogens in patients with persistent and chronic diarrhea in Nanjing, China and to further evaluate the performance and applicability of xTAG GPP in identifying pathogens in these children.

## Methods

### Sample collection

A total of 199 stool samples were prospectively collected from 199 diarrheic children mainly under 5 years of age (85.93%, Table 1), including 109 simple diarrhea and 90 secondary diarrhea (colitis, pneumonia and tumor-associated) patients who attended the Nanjing Children’s Hospital Affiliated to Nanjing Medical University (Nanjing, China). The study protocol was approved by the ethics committee of the Children’s Hospital of Nanjing Medical University (Nanjing, China). Written informed consent was obtained from the proband and their parents. One sample was received from each patient. Inclusion criteria: patients with diarrhea that presented as watery and/or loose and/or mucous and/or blood stools with ≥3 instances within a 24-h period. Patients with inflammatory bowel diseases were excluded from the study. Stool samples were sent to the Department of Microbiology for investigation. Five grams of fresh stool samples were collected into empty tubes and placed in Cary-Blair Transport Medium for bacterial culture. Stool specimens were then stored at − 80 °C until processing with multiplex PCR tests. Unqualified samples (sample volume < 5 g, swabs not preserved in Cary-Blair Transport Medium) were rejected, and resubmission was requested.

**Table 1:** Demographic and Clinical characteristics of the study subjects

Characteristics	No (%)
Demographics	
Boys	116(58.29)
Girls	83(41.71)
Age (year)	
0–1	129 (64.82)
1–5	42(21.11)
≥ 5	28(14.6)
Patients	
Out-patients	23(88.44)
In-patients	176(11.56)
Course of disease (week)	
2–8	163(85.34)
≥ 8	28(14.66)
Appearance of diarrhea	
watery /loose	139(72.78)
Mucoid / bloody	60(30.15)
Defecation frequency (Times / day)	
3–5	77 (38.69)
5–10	82(41.21)
≥ 10	40(20.10)
Use of antibiotics	
Used	151(75.88)
Unused	48(24.12)
Stool culture	
not done	50(25.13)
negative	114(57.29)
positive	35(17.59)
Diarrhea type	
Simple diarrhea^+^	109(54.77)
Secondary diarrhea*	90(45.23)

^+^ Diarrhea without colitis, pneumonia, tumor and inflammatory bowel diseases

* Diarrhea associated with colitis, pneumonia and tumor

### Routine diagnostic methods

Stool culture for *Salmonella* and *Shigella* was performed using *Salmonella*–*Shigella* agar plates and Hektoen enteric agar plates. To detect toxigenic *Clostridium difficile* A and B toxins and norovirus GI/GII real-time reverse transcription-polymerase chain reaction (RT-PCR) assays were performed on the 7500 real-time PCR platform (Applied Biosystems, Foster City, CA). Rotavirus was detected directly in stool samples with the Diagnostic Kit for Rotavirus, rapid enzyme-linked immunosorbent assay (ELISA) tests. All assays were carried out in accordance with their respective instructions. We looked for *Entamoeba histolytica* and *Giardia lamblia* by microscopic examination of fresh stools.

### Multiplex PCR and molecular diagnostic assays for the detection of 15 pathogens

Total nucleic acids were extracted from the stool samples using the NucleoSpin® Virus Kit (MACHEREY-NAGEL, Germany) according to the manufacturer’s instructions. An internal control (bacteriophage MS2) was included in each specimen to control the quality of the detection process. The RT-PCR experiments and subsequent hybridization steps were performed according to the instructions in the xTAG GPP manual. Negative and positive controls were included in all runs of the xTAG GPP assay. The data were acquired on the Luminex 200 analyzer, and data analysis was carried out using TDAS GPP version 1.11 (xTAG Data Analysis Software).

## Results

### Demographic and clinical parameters of patients with persistent and chronic diarrhea

The demographic and clinical characteristics of the 199 patients are summarized in Table 1. One hundred ninety-nine stool samples were prospectively collected from 199 diarrheic children under 5 years of age (85.93%, 171/199), with a mean age of 12.93 ± 15.86 months. The percentage of boys (58.29%, 116/199) was slightly higher than that of girls (41.71%, 83/199). There were 163 persistent cases and 28 chronic cases of diarrhea. The majority were inpatients (88.44%, 176/199) during the study period, and no deaths were reported. Of the 199 stool specimens submitted to laboratories, watery/loose stool (*n* = 139, 72.78%) was the most common type, and mucus/bloody stool was less than 30.15% (60/199).

### Pathogens detected with the xTAG GPP

In this study, we found that 145 (72.86%) of the collected 199 samples had positive results. Of these, 97 samples were positive for viruses, with rotavirus A being the most common organism detected (34.67%; 69/199). The second most abundant virus was norovirus GI/GII, which was detected in 41 patients (20.6%; 41/199). Bacterial pathogens accounted for 40.2% (80/199) of all enteropathogens; *Campylobacter* (22.11%, 44/199) was most frequently detected, and *C. difficile* toxins A/B and *Salmonella* were detected in 44 and 17 samples, respectively. Infections with *Shigella* occurred 4 times, and *E. coli* O157 was only detected once. There were three parasitic samples (1.51%); two samples were positive for *Entamoeba histolytica*, and one was positive for *Cryptosporidium*. Adenovirus 40/41, STEC, ETEC, *Giardia*, *Yersinia enterocolitica* and *Vibrio cholerae* were not detected. There were 59 coinfections (29.65% of samples) of viruses and/or bacteria and/or parasites **(**Table 2**)**. Coinfections involved 49 double infections (24.62%), 9 triple infections (4.52%) and 1 quadruple infections (0.5%). Norovirus GI/GII was found to have the highest involvement in coinfections 32 (16.08%), followed by rotavirus A (15.58%, 31/199), *Campylobacter* (12.56%, 25/199) and *C. difficile* toxin A/B (10.05%, 20/199) **(**Table 3**)**.

**Table 2:** Numbers of single and multiple infections detected by xTAG GPP

Infection	Number	No. (%)
**Single**	86	43.21%
**Double**	49	24.62%
**Triple**	9	4.52%
**Quadruple**	1	0.5%
**Multiple**	59	29.65%

**Table 3:** Pathogens detected in co-infections

pathogen	Double infection	Triple infection	Quadruple infection	Co-infection
***Rotavirus A***	23	7	1	**31(15.58%)**
***Norovirus GI/GII***	27	4	1	**32(16.08%)**
***Clostridium difficile*Toxin A/B**	14	5	1	**20(10.05%)**
***Salmonella***	8	3	0	**11(5.53%)**
***Campylotrbacter***	21	4	0	**25(12.56%)**
***Shigella***	3	1	0	**4(2.01%)**
***Escherichia coli O157***	0	1	1	**2(1.00%)**
**Entamoeba** **histolytica**	**1**	**1**	**0**	**2(1.00%)**
**Cryptosporidium**	**1**	**1**	**0**	**2(1.00%)**

### Comparison of the xTAG GPP and conventional detection methods

Among the enteropathogens that could be detected by xTAG GPP, 5 enteropathogens (STEC, ETEC, adenovirus 40/41, *Yersinia enterocolitica* and *Campylobacter*) could not be detected by routine detection methods; therefore, in this study, the specificity and sensitivity of this method for the diagnosis of these five enteropathogens were not compared. As shown in Table 4, the sensitivity was 100% for norovirus GI/GII, *C. difficile* toxin B and *Shigella*, 96.9% for rotavirus A and 33.3% for *Salmonella*. The specificity was 100% for all targets except *Entamoeba histolytica* (99.5%), *E. coli* O157 (99.0%), *Cryptosporidium* (99.0%), *Shigella* (98.0%), *Salmonella* (92.3%), rotavirus A (89.3%), norovirus GII (89.3%) and *C. difficile* toxin A/B (84.9%). Among the 10 comparable enteropathogens, 2 enteropathogens (*Giardia* and *Vibrio cholerae*) were not detected in our samples by either xTAG GPP or routine assays, so it is impossible to evaluate the sensitivity of these enteropathogens. The overall sensitivity and specificity of xTAG GPP for the diagnosis of intestinal pathogens were 96.3 and 98.2%, respectively, which were significantly higher than those of conventional detection methods. The sensitivity and specificity of this method to individual pathogens are shown in Table 4.

**Table 4:** **Comparison of xTAG GPP with the routine tests and the results of** XTAG GPP for the detection of enteric pathogens from patients with persistent and chronic diarrhea

Class	Target	GPP	No. of samples by routine tests		Performance of the xTAG GPP assay	
			+	–	Sensitivity	%Specificity
**Virus**	**Adenovirus 40/41**	+	0	0		
		–	0	199	0	100(199/199)
	**Rotavirus A**	+	63	6		
		–	2	50	96.9(63/65)	89.3(50/52)
	**Norovirus GI/GII**	+	22	19		
		–	0	158	100(22/22)	89.3(158/177)
**Bacteria**	***Salmonella***	+	2	15		
		–	4	182	33.3(2/6)	92.3(182/197)
	***Campylobacter***	+	43	0		
		–	0	199	100(43/43)	100(199/199)
	***Shigella***	+	1	3		
		–	0	195	100 (1/1)	98.5(195/198)
	***Clostridiumdifficile*Toxin A/B**	+	27	3		
		–	0	169	100(27/27)	84.9(169/172)
	**ETEC**	+	0	0		
		–	0	199	0	100(199/199)
	***Escherichiacoli*O157**	+	0	2		
		–	0	197	0	99.0(192/199)
	**STEC**	+	0	0		
		–	0	199	0	100(199/199)
	***Yersinia enterocolitica***	+	0	0		
		–	0	199	0	100(199/199)
	***Vibrio cholerae***	+	0	0		
		–	0	199	0	100(199/199)
**Parasite**	***Giardia***	+	0	0		
		–	0	199	0	100(199/199)
	***Entamoeba histolytica***	+	0	1		
		–	0	198	0	99.5(198/199)
	***Cryptosporidium***	+	0	2		
		–	0	197	0	99.0(197/199)
		+	158	51		
	**Total**	–	6	2739	96.3(158/166)	98.2(2739/2790)

### Age and sex distribution of children with enteropathogens

The prevalence of enteropathogens among sex groups was compared, 88 (75.86%) male patients and 57 (68.67%) female patients were positive for enteropathogens. The distribution of enteropathogens was similar in both boys and girls **(**Table 5**)**, with rotavirus A being the most common pathogen detected at 39.66 and 27.71%, respectively, followed by *Campylobacter* and norovirus GI/GII, and there was also no significance in coinfection (*p* > 0.05). The distributions of viruses, parasites and coinfections were similar in the three age groups (0–12 months, 12–60 months and ≥ 60 months), with *P* values of 0.73, 0.724 and 0.76, respectively **(**Table 5**)**. Rotavirus A was the most common enteropathogen in patients 0–12 months (37.9%) and 12–60 months (33.33%), while *Campylobacter* was the most frequent enteropathogen in patients ≥60 months (28.6%, 8/18). In this study, bacterial infections were the most common in the 12–60 months age group (57.1%) compared with the other age groups (33.3–46.4%).

**Table 5:** Age and sex distribution of children with enteropathogens

		Number	Negative	Virus	Bacteria	Parasite	Co-infection
	Boys	116	28	60	47	3	40
**Sex**	**Girls**	83	26	37	33	0	19
	**χ2**		1.264	0.98	0.012	2.179	2.305
	**P**		0.26	0.32	0.94	0.14	0.129
	**0–1**	129	33	70	43	2	38
**Age(year)**	**1–5**	42	11	18	24	1	14
	**≥5**	28	10	9	13	0	7
	**χ2**		2.216	5.244	7.997	0.646	0.668
	**P**		0.33	0.73	**0.018**	0.724	0.716

### Seasonal distribution of children with enteropathogens

**Fig. 1:**
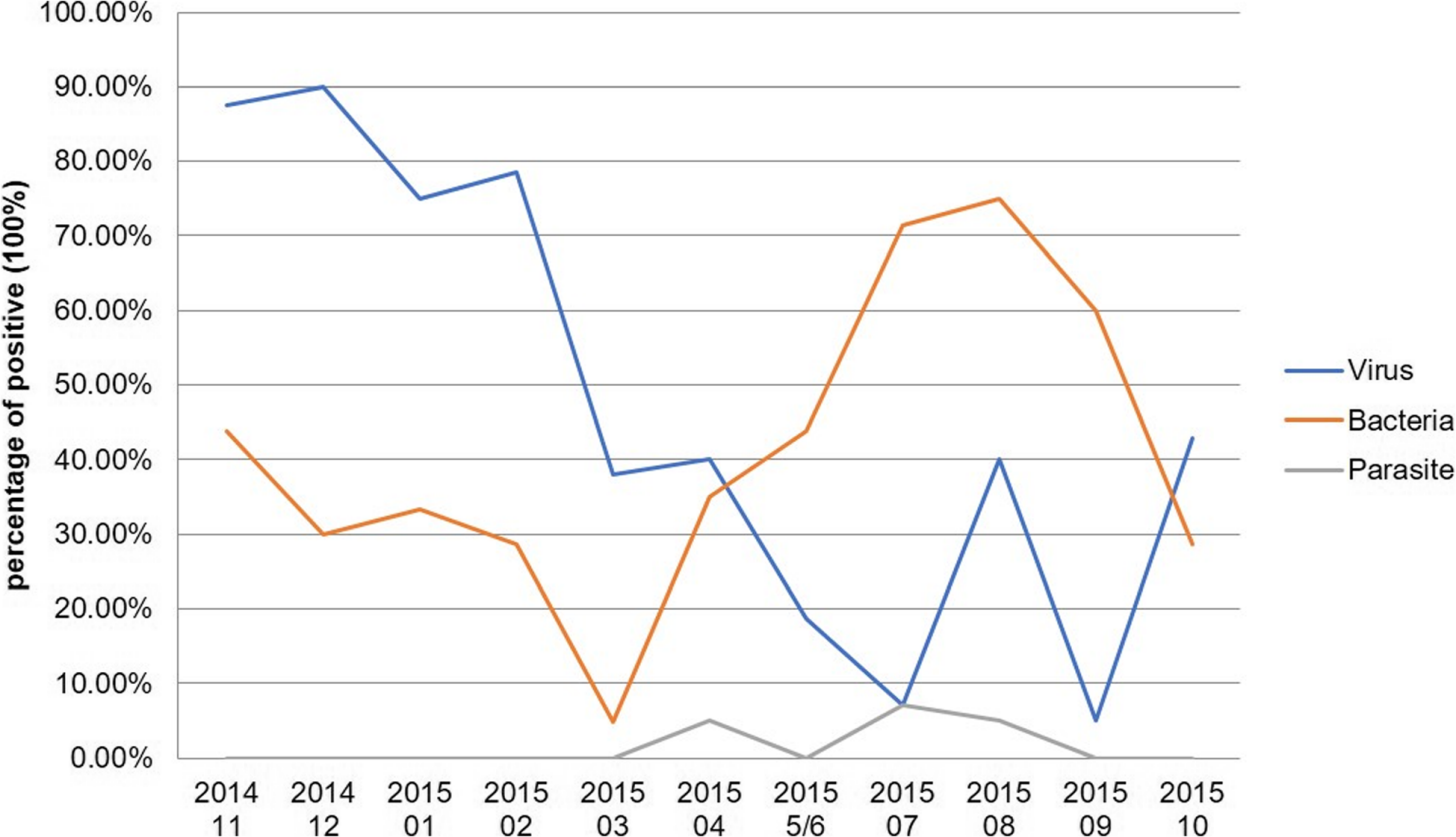
Seasonal distribution of children with enteropathogens detected by xTAG GPP assay

In this study, the seasonal curve of viral infection had a peak in the winter and a trough in the summer. Rotavirus A was the most important enteropathogen, and the infection peak occurred from November 2014 to February 2015 **(**Fig. 1**)**, with the highest proportion occurring in December 2014 (90.0%, 18/20). In contrast, bacterial agents had a peak in the summer and a trough in the winter, *Campylobacter* was the most frequent enteropathogen, and the highest proportion occurred in October 2015 (75.0%, 15/20).

## Discussion

There are few data that simultaneously describe the prevalence of bacterial and viral pathogens in children with persistent and chronic diarrhea in China. In our research, persistent and chronic diarrhea were related to sex and age and commonly existed in boys, especially in children under 2 years old. In our study, 129 patients ages 0–1 years old (64.82%), this was similar to the research showing that the morbidity age was 4 months to 1 year [3]. Patients who had watery and/or loose stool predominated more than patients who had mucoid/bloody stool, even though most samples were collected in the winter.

In this study, the overall sensitivity and specificity of xTAG GPP were 96.3 and 98.2%, respectively, which were more efficient than routine detection methods. At the same time, there were significant differences in single or mixed intestinal enteropathogen infection (*P* < 0.001) (Table 4). In the present study, the xTAG GPP method efficiently detected infection in approximately 59 (29.65%) out of 199 children and showed multiple positive results (coinfection); this figure is higher than the positive result, which was relatively high and also detected by xTAG GPP, in a previous study by *Deng J* et al. [13]. Norovirus GI/GII was found to have the highest involvement in coinfections in our study.

In our study, rotavirus A was the most common pathogen in children with chronic diarrhea in the spring and autumn, followed by norovirus. Previous reports have shown that rotavirus A is the most common virus that causes diarrhea in children [15]. Moreover, norovirus is an important cause of diarrhea in adults and children [16]. This result was similar to other studies conducted previously in China [17–19] as well as other countries prior to the introduction of rotavirus vaccination [20, 21].

In our study, 4 samples with a positive *Salmonella* culture had no positive results in xTAG GPP, indicating false-negative results. This phenomenon is consistent with some previously reported results [13, 22]. The cause of the failure of the *Salmonella* pathogen requires further sequence analysis or qPCR assay investigation. Special attention should be paid to the occurrence of *Campylobacter* because the detection of this pathogen is rarely requested in patients with diarrhea; however, in our study, we detected a high frequency of 25% (43/199) for *Campylobacter*.

## Conclusions

In conclusion, our research shows that xTAG GPP has very good sensitivity and specificity in detecting pathogens associated with persistent and chronic diarrhea. This method can shorten the detection time and reduce false-negative diagnostics, identify the cause of infection more quickly and accurately, provide a basis for accurate follow-up clinical treatment and improve the prognosis of the disease. However, the number of samples in this experiment is limited, and some pathogens have no positive samples (*Giardia* and *Vibrio cholerae*), so it is impossible to compare the results from different methods.

## Data Availability

All data generated or analysed during this study are included in this published article.

## Abbreviations

xTAG GPP: xTAG gastrointestinal pathogen panel
ETEC: Enterotoxigenic *Escherichia coli*
STEC: Shiga-like Toxin producing E.coli
RT-PCR: Realtime reverse transcription-polymerase chain reaction

## References

[1] Fischer Walker CL, Perin J, Aryee MJ, Boschi-Pinto C, Black RE (2012). Diarrhea incidence in low- and middle-income countries in 1990 and 2010: a systematic review. BMC Public Health.

[2] Kotloff KL, Blackwelder WC, Nasrin D, Nataro JP, Farag TH, van Eijk A, Adegbola RA, Alonso PL, Breiman RF, Faruque AS, Saha D, Sow SO, Sur D, Zaidi AK, Biswas K, Panchalingam S, Clemens JD, Cohen D, Glass RI, Mintz ED, Sommerfelt H, Levine MM (2012). The global enteric multicenter study (GEMS) of diarrheal disease in infants and young children in developing countries: epidemiologic and clinical methods of the case/control study. Clinical infectious diseases : an official publication of the Infectious Diseases Society of America.

[3] Lima AA, Guerrant RL (1992). Persistent diarrhea in children: epidemiology, risk factors, pathophysiology, nutritional impact, and management. Epidemiol Rev.

[4] Victora CG, Huttly SR, Fuchs SC, Barros FC, Garenne M, Leroy O, Fontaine O, Beau JP, Fauveau V, Chowdhury HR (1993). International differences in clinical patterns of diarrhoeal deaths: a comparison of children from Brazil, Senegal, Bangladesh, and India. J Diarrhoeal Dis Res.

[5] Bhutta ZA, Nelson EA, Lee WS, Tarr PI, Zablah R, Phua KB, Lindley K, Bass D, Phillips A, Persistent Diarrhea Working G (2008). Recent advances and evidence gaps in persistent diarrhea. J Pediatr Gastroenterol Nutr.

[6] Lee KS, Kang DS, Yu J, Chang YP, Park WS (2012). How to do in persistent diarrhea of children?: concepts and treatments of chronic diarrhea. Pediatric gastroenterology, hepatology & nutrition.

[7] Vocale C, Rimoldi SG, Pagani C, Grande R, Pedna F, Arghittu M, Lunghi G, Maraschini A, Gismondo MR, Landini MP, Torresani E, Topin F, Sambri V (2015). Comparative evaluation of the new xTAG GPP multiplex assay in the laboratory diagnosis of acute gastroenteritis. Clinical assessment and potential application from a multicentre Italian study International journal of infectious diseases : IJID : official publication of the International Society for Infectious Diseases.

[8] Guerrant RL, Van Gilder T, Steiner TS, Thielman NM, Slutsker L, Tauxe RV, Hennessy T, Griffin PM, DuPont H, Sack RB, Tarr P, Neill M, Nachamkin I, Reller LB, Osterholm MT, Bennish ML, Pickering LK, Infectious Diseases Society of A (2001). Practice guidelines for the management of infectious diarrhea. Clinical infectious diseases : an official publication of the Infectious Diseases Society of America.

[9] Liu J, Gratz J, Maro A, Kumburu H, Kibiki G, Taniuchi M, Howlader AM, Sobuz SU, Haque R, Talukder KA, Qureshi S, Zaidi A, Haverstick DM, Houpt ER (2012). Simultaneous detection of six diarrhea-causing bacterial pathogens with an in-house PCR-luminex assay. J Clin Microbiol.

[10] Maes B, Hadaya K, de Moor B, Cambier P, Peeters P, de Meester J, Donck J, Sennesael J, Squifflet JP (2006). Severe diarrhea in renal transplant patients: results of the DIDACT study. Am J Transplant Off J Am Soc Transplant Am Soc Transplant Surg.

[11] Higgins RR, Beniprashad M, Cardona M, Masney S, Low DE, Gubbay JB (2011). Evaluation and verification of the Seeplex Diarrhea-V ACE assay for simultaneous detection of adenovirus, rotavirus, and norovirus genogroups I and II in clinical stool specimens. J Clin Microbiol.

[12] Malecki M, Schildgen V, Kamm M, Mattner F, Schildgen O (2012). Rapid screening method for multiple gastroenteric pathogens also detects novel enterohemorrhagic Escherichia coli O104:H4. Am J Infect Control.

[13] Deng J, Luo X, Wang R, Jiang L, Ding X, Hao W, Peng Y, Jiang C, Yu N, Che X (2015). A comparison of Luminex xTAG(R) gastrointestinal pathogen panel (xTAG GPP) and routine tests for the detection of enteropathogens circulating in southern China. Diagn Microbiol Infect Dis.

[14] Claas EC, Burnham CA, Mazzulli T, Templeton K, Topin F (2013). Performance of the xTAG(R) gastrointestinal pathogen panel, a multiplex molecular assay for simultaneous detection of bacterial, viral, and parasitic causes of infectious gastroenteritis. J Microbiol Biotechnol.

[15] Wilhelmi de Cal I, Mohedano del Pozo RB, Sanchez-Fauquier A (2008) [Rotavirus and other viruses causing acute childhood gastroenteritis]. Enfermedades infecciosas y microbiologia clinica 26 Suppl 13:61–65. 10.1157/13128782 PMC7130379 19100169

[16] Glass RI, Parashar UD, Estes MK (2009). Norovirus gastroenteritis. N Engl J Med.

[17] Chen Y, Li Z, Han D, Cui D, Chen X, Zheng S, Yu F, Liu J, Lai S, Yan Y, Lin Z, Shi Z, Wu T, Li L, Yang W (2013). Viral agents associated with acute diarrhea among outpatient children in southeastern China. Pediatr Infect Dis J.

[18] Yu J, Jing H, Lai S, Xu W, Li M, Wu J, Liu W, Yuan Z, Chen Y, Zhao S, Wang X, Zhao Z, Ran L, Wu S, Klena JD, Feng L, Li F, Ye X, Qiu Y, Wang X, Yu H, Li Z, Yang W (2015). Etiology of diarrhea among children under the age five in China: results from a five-year surveillance. The Journal of infection.

[19] Lou JT, Xu XJ, Wu YD, Tao R, Tong MQ (2011). Epidemiology and burden of rotavirus infection among children in Hangzhou, China. Journal of clinical virology : the official publication of the Pan American Society for Clinical Virology.

[20] Podkolzin AT, Fenske EB, Abramycheva NY, Shipulin GA, Sagalova OI, Mazepa VN, Ivanova GN, Semena AV, Tagirova ZG, Alekseeva MN, Molochny VP, Parashar UD, Vinje J, Maleev VV, Glass RI, Pokrovsky VI (2009). Hospital-based surveillance of rotavirus and other viral agents of diarrhea in children and adults in Russia, 2005-2007. J Infect Dis.

[21] (2000) Report of the study of infectious intestinal disease in England. Communicable disease report CDR weekly 10 (51):457. 11191030

[22] Mengelle C, Mansuy JM, Prere MF, Grouteau E, Claudet I, Kamar N, Huynh A, Plat G, Benard M, Marty N, Valentin A, Berry A, Izopet J (2013). Simultaneous detection of gastrointestinal pathogens with a multiplex Luminex-based molecular assay in stool samples from diarrhoeic patients. Clinical microbiology and infection : the official publication of the European Society of Clinical Microbiology and Infectious Diseases.

